# Reactive Oxygen Species in Cardiovascular Calcification: Role of Medicinal Plants

**DOI:** 10.3389/fphar.2022.858160

**Published:** 2022-03-15

**Authors:** Yu Qiao

**Affiliations:** King’s College London, London, United Kingdom

**Keywords:** cardiovascular calcification, experimental evidence, reactive oxygen species, review, therapy, medicinal plants

## Abstract

Cardiovascular calcification, including vascular calcification and calcific aortic valve disease (CAVD), is a serious worldwide health problem, especially in older adults. The mechanisms underlying cardiovascular calcifications are complex and multifactorial. An increase in reactive oxygen species (ROS) and oxidative stress play important roles in the initiation and development of cardiovascular calcification. This mini-review summarizes the recent evidence that supports the association of ROS with vascular calcification and CAVD and discusses the role of medicinal plants for the prevention and treatment of cardiovascular calcification.

## 1 Introduction

Cardiovascular calcification is a prevalent and common pathological factor in several cardiovascular diseases, including vascular calcification and calcific aortic valve diseases (CAVD). The early stage of vascular calcification is characterized by the formation of a calcified plaque, stiffness of vascular walls, and a decrease in vascular compliance. Meanwhile, the advanced stage shows inflammatory infiltration, fibrosis, and formation of an atheromatous plaque (Li et al., 2021). At present, oxidative stress is one of the known significant factors contributing to cardiovascular calcification development. Reactive oxygen species (ROS) excessively accumulate intracellularly, leading to oxidative stress. Increased oxidative stress might play significant roles in the initiation and progression of cardiovascular calcification and the conversion of vascular smooth muscle cells into osteoblast-like phenotype (Lee et al., 2020). Emerging evidence indicates that other sources such as mitochondria, uncoupled eNOS, and xanthine oxidases also contribute to several risk factors related to cardiovascular calcification (Hu et al., 2021). However, the precise mechanisms and associations in cardiovascular calcification development are not well understood. Studies have shown that medicinal plants and their active ingredients could significantly improve vascular calcification. Hence, the role of ROS and medicinal plants in vascular calcification will be reviewed.

## 2 Reactive Oxygen Species in Cardiovascular Calcification

### 2.1 The Sources of Reactive Oxygen Species in Cardiovascular Calcification

#### 2.1.1 NADPH Oxidase Protein

NADPH oxidases are the primary sources of ROS in the vascular wall, which play important roles in the pathophysiology of the cardiovascular system. NOX2 and NOX4 are major isoforms expressed in the heart and vessels (Zhang et al., 2010). Under the condition of degenerative aortic valve stenosis, the expression of NOX2 isoform was increased around the calcifying foci in the stenotic aortic valve, where NOX4 is also upregulated during calcification (Liberman et al., 2008). An increasing level of hydrogen peroxide in the aortic valve is possibly related to the NADPH oxidase activity. Increased NOX2 and NOX4 levels and increased oxidative stress might contribute to the calcification process.

#### 2.1.2 Mitochondria

Mitochondrial ROS is associated with vascular calcification (Zhao et al., 2011). Accumulation of oxidants could result in mitochondrial dysfunction, consequently leading to vascular calcification (Cui et al., 2017). Overexpression of p66Shc leads to an increase in ROS production, which results in the stimulation of the dysfunctional phenotype in endothelial cells, eventually triggering mitochondrial apoptosis leading to calcification (Pagnin et al., 2005). Therefore, targeting p66Shc can be a potential therapy to reduce mitochondrial oxidative stress.

#### 2.1.3 Uncoupled eNOS

Under normal circumstances, eNOS produces nitric oxide. However, eNOS could also generate hydrogen peroxide and nitric oxide when uncoupled. Uncoupling of eNOS is induced either by the endogenous competitive inhibitor asymmetric dimethylarginine or reduction of BH4 bioavailability (Incalza et al., 2018). eNOS uncoupling plays a significant role in elevated endothelial oxidative stress and consequently triggers endothelial dysfunction, which might contribute to aortic valve calcification by degradation of endothelial protection (Farrar et al., 2015).

#### 2.1.4 Xanthine Oxidase

Under inflammatory conditions, the reductase form could switch to oxidase form (xanthine oxidase, XO) (Incalza et al., 2018). XO-induced ROS predominantly contributes to ischemia- or reperfusion injury-induced oxidative stress. Human atherosclerotic plaque rupture is associated with vascular calcification, where there is an elevation of both the endothelial XO and plasma XO, indicating the contribution of XO to human atherosclerosis (Förstermann et al., 2017). Moreover, XDH switches to XO when released to the blood circulation, leading to further ROS generation and endothelial dysfunction by binding XO with sulfated glycosaminoglycans.

### 2.2 ROS Signaling Pathways

Oxidative stress might also induce the activation of a few pathways, which have beneficial effects. Although emerging evidence demonstrates the connection between ROS and cardiovascular calcification, the precise mechanism remains incompletely understood. However, recent studies suggest some potential mechanisms.

#### 2.2.1 RUNX2

RUNX2 is induced by AKT signaling via hydrogen peroxide activation of PI3K (Chen et al., 2019). A mouse model experiment indicates that when hydrogen peroxide level is 0.1–0.4 mmol/L, trans-differentiation of smooth muscle cells into osteogenic phenotype occurs in a dependent manner, and the RUNX2 expression level shows a significantly increasing trend (Leopold, 2015). Bone-related transcription factors, such as RUNX2, Msx2, and sox9, were found to be upregulated in the calcified blood vessel. Hydrogen peroxide-induced activation of PI3K/AKT signaling results in upregulation of RUNX2 and, thus, calcification of vascular smooth muscle cells (Byon et al., 2008).

#### 2.2.2 JNK Pathway

JNK plays an important role in inflammation and apoptosis, which is involved in the pathophysiology of coronary heart disease, ischemia-reperfusion injury, chronic inflammation, and neurodegeneration. ROS-induced activation of JNK could activate both the intrinsic and extrinsic apoptosis pathways. ROS can activate JNK by activation of ASK1 and trigger the translocation of FoxO1 into the nucleus, thus leading to the upregulation of FoxO1 expression and consequently the regulation of oxidative stress (Zhang et al., 2019). JNK/Sab/Src signaling pathway could inhibit the electron transport chain, leading to more generation of mitochondrial ROS to induce apoptosis. However, the type of ROS that can activate the JNK signaling pathway is still unknown.

#### 2.2.3 TLR4/NF-κB/Ceramide Signaling

Oxidized low-density lipoprotein (Ox-LDL) acts as a significant biomarker of oxidative stress, which is an important risk factor for osteogenic differentiation of vascular smooth muscle cells. Ox-LDL induces upregulation of TLR4 expression, triggering the activation of NF-κB, which is important in regulating inflammation acting downstream of TLR4 (Song et al., 2017). Inhibition of the NF-κB signaling pathway prevents further increase of ceramide, which indicates ceramide may act downstream of NF-κB signaling. Collectively, oxidative stress-related Ox-LDL could trigger vascular calcification *via* TLR4/NF-κB/ceramide signaling pathway (Song et al., 2017). These findings highlight the importance of NF-κB in vascular calcification development.

### 2.3 Mechanism of Reactive Oxygen Species-Mediated Cardiovascular Calcification

#### 2.3.1 Apoptosis

ROS-induced apoptosis is another potential mechanism leading to valvular and vascular calcification, and accumulation of apoptotic bodies may form nucleation sites for calcium deposition (Redza-Dutordoir and Averill-Bates, 2016). Endoplasmic reticulum stress- (ERS-) induced apoptosis is a prevalent pathology in atherosclerosis, type II diabetes, and chronic kidney diseases. ERS triggers aortic calcification in rat models via induction of vascular smooth muscle cell apoptosis (Shi et al., 2019). ERS could induce the synthesis of NADPH oxidase, which induces further apoptosis (Zhou et al., 2021). The relationship between ROS and the endoplasmic reticulum needs further exploration.

#### 2.3.2 Inflammation

Calcifying aortic valve disease is thought to be caused by inflammation and immune response. However, in the calcified valve or vascular tissues, a large number of macrophages and lymphocytes infiltration are found (Incalza et al., 2018). NLRP3 inflammasome is regulated by ROS, and under cellular stress, NLRP could activate caspase-1 and process cytoplasmic targets. Inhibition of mitophagy would result in ROS accumulation, thus activating NLRP3 (Xu. et al., 2020). Activation of NLRP3 can trigger the maturation of pro-inflammatory cytokines such as IL-1β. Pro-inflammatory cytokines could also induce ROS generation (Kelley et al., 2019). ROS might trigger cardiovascular calcification by inducing pro-inflammatory cytokines (Pawade et al., 2015).

#### 2.3.3 Autophagy

The role of autophagy in vascular calcification is complex. Autophagy inhibits vascular calcification by reducing electrolyte imbalance. For example, Fe^3+^ could reduce vascular calcification by inducing increased autophagy (Ciceri et al., 2016). Conversely, autophagy can promote vascular calcification by activating different pathways. Autophagy induces calcification by increasing stromal vesicle release (Phadwal et al., 2020). The interaction between autophagy and oxidative stress plays an important role in vascular calcification. 7-KC induces oxidative stress and accelerates vascular calcification by inhibiting autophagosome and lysosome dysfunction (Sudo et al., 2015). Inhibition of autophagy activates oxidative stress and promotes vascular calcification. In conclusion, autophagy plays different roles in vascular calcification through different pathways. Regulation of autophagy to reduce the severity of vascular calcification will provide new ideas for studying the vascular calcification mechanism and clinical prevention and treatment.

## 3 Prevention and Treatment for Cardiovascular Calcification

Regulation of ROS generation and balance of ROS level is significant in preventing cardiovascular diseases. Recent studies have demonstrated that modern drugs might have some beneficial impacts on the treatment of cardiovascular calcification, and the role of medicinal plants in cardiovascular calcification also attracted broad attention.

### 3.1 Modern Medicine in Cardiovascular Calcification

Bisphosphonates are inhibitors for osteoclast-mediated bone resorption. It has shown an effective reduction in the calcified vasculature and the aortic valve. The nitrogen-containing bisphosphonates have been indicated to have significant anti-calcific effects in the vasculature by acting as inorganic pyrophosphate analogs (Pawade et al., 2015). Moreover, bisphosphonates reduce the differentiation of aortic valve myofibroblasts into osteogenic-like phenotypes (Elmariah et al., 2010). Collectively, these effects indicated that bisphosphonates might be an effective therapy in cardiovascular calcification-induced aortic stenosis.

Denosumab is an antibody that prevents RANKL from binding to RANK. Clinical data shows that “in 7,868 post-menopausal women with osteoporosis, denosumab increased bone mineral density and reduced vertebral fracture by 68% over a three-year period” (Pawade et al., 2015). These data indicate that denosumab has an important role in regulating the OPG/RANK/RANKL system in vascular and aortic valve calcification (Pawade et al., 2015).

The statin and angiotensin-converting enzyme (ACE) inhibitors might halve the progression of aortic valve stenosis (Kleinauskienė and Jonkaitienė., 2018; Andersson and Abdulla, 2017). In addition, statin and ACE could reduce oxidative stress (Daiber et al., 2021) and, hence, be a potential therapeutic option that targets oxidative stress-induced early-stage calcification.

### 3.2 Medicinal Plants in Cardiovascular Calcification

In recent years, much attention has been paid to the role of medicinal plants in vascular calcification. We summarize the progress of medicinal plants or their extracts for vascular calcification.

#### 3.2.1 *Ginkgo biloba* L.

Studies have shown that *Ginkgo biloba* L. extract (GBE) can reduce vascular calcification (Xu et al., 2015). β-Glycerophosphate induce VSMC calcification by activating the Wnt/β-catenin signaling pathway. GBE could alleviate the VSMC calcification induced by β-glycerophosphate through inhibition of the Wnt/β-catenin signaling pathway (Wang et al., 2019). Studies suggest that GBE not only significantly reduced calcium deposition in rat aortic smooth muscle cells induced by β-glycerophosphate but also inhibited osteogenic trans-differentiation, which may be related to the decrease of alkaline phosphatase expression, NF-κB activity, and reactive oxygen species production in vascular smooth muscle cells (Li et al., 2016). Another study showed that GBE could improve warfarin-induced aortic valve calcification by inhibiting the BMP2-mediated SMad1/5/Runx2 signaling pathway (Liu et al., 2021). GBE may be a potential medicinal plant for vascular calcification.

#### 3.2.2 Celastrol

Celastrol is an extract of the *Tripterygium wilfordii* plant, a traditional Chinese medicine, which belongs to the pentacyclic triterpenoids. Recent studies have shown that celastrol could exhibit an inhibition effect on NOX activity. Celastrol could induce protection effects on inflammation (Hu et al., 2017), fibrosis (Guo et al., 2016), and atherosclerosis (Gu et al., 2013), which are risk factors for ROS-induced cardiovascular calcification. Interestingly, celastrol could inhibit NADPH oxidase isoforms (Jaquet et al., 2011). Celastrol reduces aortic valve calcification by inhibiting NOX2 in valve interstitial cells, which is associated with inhibiting the NOX2-mediated glycogen synthase kinase 3β/β-catenin pathway in calcified aortic valve disease (Liu et al., 2019a). Therefore, celastrol might be a new potential therapy for cardiovascular calcification by inhibiting NOX activities.

#### 3.2.3 Quercetin

Quercetin, a flavonoid found in vegetables, tea, and fruits, has bioactive properties, including antioxidant, anti-inflammatory, and anti-apoptotic (Yang et al., 2020). Quercetin interferes with adenine-induced aortic calcification in rats and calcified vascular smooth muscle cells induced by inorganic salts. The results showed that quercetin reduced apoptosis of vascular smooth muscle cells by blocking oxidative stress and inhibiting mitochondrial fission quercetin, and quercetin also significantly improved adenine-induced aortic calcification in rats (Cui et al., 2017).

#### 3.2.4 Curcumin

Curcumin is the main active component in *Curcuma longa* L*.*, which can inhibit inflammation and resist oxidation. Studies have shown that curcumin inhibits osteogenic differentiation of human aortic valve stromal cells by activating NF-*k*B/AKT/ERK signaling pathway (Zhou et al., 2020). Other studies suggested that curcumin might inhibit apoptosis and calcification of vascular smooth muscle cells by inhibiting JNK/Bax signaling pathway (Hou el at., 2016). Taken together, curcumin could be used to prevent and treat vascular calcification potentially.

#### 3.2.5 Resveratrol

Resveratrol (trans-3,4′,5-trihydroxystibene) is a natural antioxidant, naturally occurring polyphenolic compound found in many plants, such as grapes, red wine, and mulberries. Researchers have established a vascular calcification model induced by stimulation of rat vascular smooth muscle cells with β-glycophosphate and investigated the effect of resveratrol on vascular calcification. Results showed that resveratrol prevents calcium deposition and mitochondrial dysfunction induced by vascular calcification by involving SIRtuin-1 and Nrf2 (Zhang et al., 2016). Resveratrol has therapeutic effects on various diseases. The degree of aortic atherosclerosis and vascular calcification in uremic mice was reduced after resveratrol supplementation, and the vascular health of mice was promoted (Tomayko et al., 2014). In conclusion, resveratrol supplements are beneficial for vascular health.

#### 3.2.6 Puerarin

Puerarin is a kind of isoflavone compound, which is the main bioactive ingredient extracted from *Pueraria* roots and has anti-inflammatory, antioxidant, and anti-apoptosis effects. Studies have shown that puerarin attenuates osteoblast differentiation of vascular smooth muscle cells through the ER/PI3K-Akt signaling pathway (Lu et al., 2014). Puerarin might reduce IL-1β by targeting NLRP3/caspase-1/IL-1β and NF-κB signaling pathways and inhibit the production of reactive oxygen species, ultimately delaying vascular calcification (Liu et al., 2019b). Puerarin could protect blood vessels and has great potential in the clinical treatment of cardiovascular calcification.

#### 3.2.7 Astragaloside

Astragaloside, extracted from *Astragalus mongholicus* Bunge, could protect blood vessels. Arterial calcification can be inhibited by promoting the autophagy of vascular smooth muscle cells, whose loss of autophagy leads to atherosclerosis. Evidence has shown that astragaloside IV could attenuate autophagy and mineralization of VSMCs in atherosclerosis, which may be related to H19 overexpression and DUSP5 inhibition (Song et al., 2019).

#### 3.2.8 Ginsenosides

Ginsenosides, isolated from the dried roots of *Panax ginseng* C.A.Mey., could be used to protect blood vessels from inflammation and oxidative stress, among others. Ginsenosides play an important role in treating cardiovascular diseases based on atherosclerosis and inhibiting the pathogenesis of arterial calcification (Xue et al., 2021). Ginsenoside Rg1 could enhance the expression of autophagy-related proteins and reduce the expression of apoptotic proteins through the AMPK/mTOR pathway, thereby reducing apoptosis, enhancing autophagy, and helping to slow down vascular calcification and delay the emergence of atherosclerosis (Yang et al., 2018).

#### 3.2.9 Emodin

Emodin, which can be isolated from several Chinese herbs, including *Rheum palmatum* L. and *Polygonum cuspidatum*, has been reported to possess numerous bioactivities (e.g., diuretic, anti-inflammatory, antiviral) and inhibits oxidative stress (Li et al., 2020). Emodin could attenuate atherosclerosis, and researchers further focused on whether emodin could attenuate vascular calcification. To investigate the role of emodin in vascular calcification, researchers induced aortic valvular calcification in mice with vitamin D and treated them with emodin. Emodin inhibited calcium levels in serum and valves and reduced calcium accumulation in the aortic valvular by inhibiting the AKT/FOXO1 signaling pathway (Luo et al., 2022). Evidence suggested that emodin-induced oxidative inhibition of mitochondrial function contributes to ER-associated apoptosis mediated by BiP/IRE1α/CHOP signaling pathway (Qiu et al., 2021). Emodin could alleviate calcium-related aortic valvular calcification, providing new insights into therapeutic strategies for clinical valve calcification.

## 4 Perspective

ROS could interact with other mechanisms and promote each other in the initiation and progression of cardiovascular calcification. Although intracellular signal transduction of ROS has been widely investigated, the precise targets and mechanisms of ROS in cardiovascular calcification and ossification remain incompletely understood. The investigation of the ROS mechanism, targeting different types of ROS, and its targets are warranted. In the future, the development of a new type of cell organelle targeted probes and antioxidants might contribute to a better understanding of the roles of ROS in aerobic/anaerobic respiration, fatty acid synthesis, and modification after protein translation and transcription. These techniques could also contribute to research by understanding the influence of pathological changes of ROS-induced inflammation and cardiovascular calcification. Additionally, the pathogenesis of cardiovascular calcification is complex. Understanding tissue-specific oxidative and reductive signals would contribute to exploring new therapies and technologies. Studies have shown that active ingredients of medicinal plants could significantly improve vascular calcification, suggesting that medicinal plants may be a potential treatment for vascular calcification. However, there are few relevant research articles and a lack of high-quality clinical evidence-based studies. We should further explore the role of medicinal plants in the prevention and treatment of vascular calcification.

## References

[B1] Andersson C. Abdulla J. (2017). Is the Use of Renin-Angiotensin System Inhibitors in Patients with Aortic Valve Stenosis Safe and of Prognostic Benefit? A Systematic Review and Meta-Analysis. Eur. Heart J. Cardiovasc. Pharmacother. 3 (1), 21–27. 10.1093/ehjcvp/pvw027 27615013

[B2] Byon C. H. Javed A. Dai Q. Kappes J. C. Clemens T. L. Darley-Usmar V. M. (2008). Oxidative Stress Induces Vascular Calcification through Modulation of the Osteogenic Transcription Factor Runx2 by AKT Signaling. J. Biol. Chem. 283 (22), 15319–15327. 10.1074/jbc.M800021200 18378684 PMC2397455

[B3] Chen P. C. Liu J. F. Fong Y. C. Huang Y. L. Chao C. C. Tang C. H. (2019). CCN3 Facilitates Runx2 and Osterix Expression by Inhibiting miR-608 through PI3K/Akt Signaling in Osteoblasts. Int. J. Mol. Sci. 20 (13), 3300. 10.3390/ijms20133300 PMC6651805 31284378

[B4] Ciceri P. Elli F. Braidotti P. Falleni M. Tosi D. Bulfamante G. (2016). Iron Citrate Reduces High Phosphate-Induced Vascular Calcification by Inhibiting Apoptosis. Atherosclerosis 254, 93–101. 10.1016/j.atherosclerosis.2016.09.071 27716569

[B5] Cui L. Li Z. Chang X. Cong G. Hao L. (2017). Quercetin Attenuates Vascular Calcification by Inhibiting Oxidative Stress and Mitochondrial Fission. Vascul Pharmacol. 88, 21–29. 10.1016/j.vph.2016.11.006 27932069

[B6] Daiber A. Steven S. Euler G. Schulz R. (2021). Vascular and Cardiac Oxidative Stress and Inflammation as Targets for Cardioprotection. Curr. Pharm. Des. 27 (18), 2112–2130. 10.2174/1381612827666210125155821 33550963

[B7] Elmariah S. Delaney J. A. O'Brien K. D. Budoff M. J. Vogel-Claussen J. Fuster V. (2010). Bisphosphonate Use and Prevalence of Valvular and Vascular Calcification in Women MESA (The Multi-Ethnic Study of Atherosclerosis). J. Am. Coll. Cardiol. 56 (21), 1752–1759. 10.1016/j.jacc.2010.05.050 21070928 PMC3004769

[B50] Farrar E. J. Huntley G. D. Butcher J. (2015). Endothelial-Derived Oxidative Stress Drives Myofibroblastic Activation and Calcification of the Aortic Valve. Plos One 10 (4), e0123257. 25874717 10.1371/journal.pone.0123257 PMC4395382

[B8] Förstermann U. Xia N. Li H. (2017). Roles of Vascular Oxidative Stress and Nitric Oxide in the Pathogenesis of Atherosclerosis. Circ. Res. 120 (4), 713–735. 28209797 10.1161/CIRCRESAHA.116.309326

[B9] Gu L. Bai W. Li S. Zhang Y. Han Y. Gu Y. (2013). Celastrol Prevents Atherosclerosis via Inhibiting LOX-1 and Oxidative Stress. PLoS ONE 8 (6), e65477. 10.1371/journal.pone.0065477 23799016 PMC3684610

[B52] Guo X. Xue M. Li C. Yang W. Wang S. Ma Z. (2016). Protective Effects of Triptolide on TLR4 Mediated Autoimmune and Inflammatory Response Induced Myocardial Fibrosis in Diabetic Cardiomyopathy. J. Ethnopharmacol. 193, 333–344. 27558948 10.1016/j.jep.2016.08.029

[B10] Hou M. Song Y. Li Z. Luo C. Ou J. S. Yu H. (2016). Curcumin Attenuates Osteogenic Differentiation and Calcification of Rat Vascular Smooth Muscle Cells. Mol. Cel Biochem 420 (1-2), 151–160. 10.1007/s11010-016-2778-y 27502306

[B11] Hu C. T. Shao Y. D. Liu Y. Z. Xiao X. Cheng Z. B. Qu S. L. (2021). Oxidative Stress in Vascular Calcification. Clin. Chim. Acta 519, 101–110. 10.1016/j.cca.2021.04.012 33887264

[B12] Hu M. Luo Q. Alitongbieke G. Chong S. Xu C. Xie L. (2017). Celastrol-Induced Nur77 Interaction with TRAF2 Alleviates Inflammation by Promoting Mitochondrial Ubiquitination and Autophagy. Mol. Cel 66 (1), 141.e6–153.e6. 10.1016/j.molcel.2017.03.008 PMC5761061 28388439

[B13] Incalza M. A. D'Oria R. Natalicchio A. Perrini S. Laviola L. Giorgino F. (2018). Oxidative Stress and Reactive Oxygen Species in Endothelial Dysfunction Associated with Cardiovascular and Metabolic Diseases. Vascul Pharmacol. 100, 1–19. 10.1016/j.vph.2017.05.005 28579545

[B14] Jaquet V. Marcoux J. Forest E. Leidal K. G. McCormick S. Westermaier Y. (2011). NADPH Oxidase (NOX) Isoforms Are Inhibited by Celastrol with a Dual Mode of Action. Br. J. Pharmacol. 164 (2b), 507–520. 10.1111/j.1476-5381.2011.01439.x 21501142 PMC3188888

[B15] Kelley N. Jeltema D. Duan Y. He Y. (2019). The NLRP3 Inflammasome: An Overview of Mechanisms of Activation and Regulation. Int. J. Mol. Sci. 20 (13), 3328. 10.3390/ijms20133328 PMC6651423 31284572

[B16] Kleinauskienė R. Jonkaitienė R. (2018). Degenerative Aortic Stenosis, Dyslipidemia and Possibilities of Medical Treatment. Medicina (Kaunas, Lithuania) 54 (2), 24. 10.3390/medicina54020024 PMC6037252 30344255

[B17] Lee S. J. Lee I. K. Jeon J. H. (2020). Vascular Calcification-New Insights into its Mechanism. Int. J. Mol. Sci. 21 (8), 2685. 10.3390/ijms21082685 PMC7216228 32294899

[B18] Leopold J. A. (2015). Vascular Calcification: Mechanisms of Vascular Smooth Muscle Cell Calcification. Trends Cardiovasc. Med. 25 (4), 267–274. 10.1016/j.tcm.2014.10.021 25435520 PMC4414672

[B19] Li E. G. Tian J. Xu Z. H. (2016). Effects of Gingko Biloba Extract (EGb 761) on Vascular Smooth Muscle Cell Calcification Induced by β-glycerophosphate. Ren. Fail. 38 (4), 552–557. 10.3109/0886022X.2016.1148724 26908182

[B20] Li Q. Gao J. Pang X. Chen A. Wang Y. (2020). Molecular Mechanisms of Action of Emodin: as an Anti-cardiovascular Disease Drug. Front. Pharmacol. 11, 559607. 10.3389/fphar.2020.559607 32973538 PMC7481471

[B21] Li W. Su S. A. Chen J. Ma H. Xiang M. (2021). Emerging Roles of Fibroblasts in Cardiovascular Calcification. J. Cel Mol Med 25 (4), 1808–1816. 10.1111/jcmm.16150 PMC7882970 33369201

[B22] Liberman M. Bassi E. Martinatti M. K. Lario F. C. Wosniak J. Pomerantzeff P. M. (2008). Oxidant Generation Predominates Around Calcifying Foci and Enhances Progression of Aortic Valve Calcification. Arterioscler Thromb. Vasc. Biol. 28 (3), 463–470. 10.1161/ATVBAHA.107.156745 18162610

[B23] Liu H. Wang L. Pan Y. Wang X. Ding Y. Zhou C. (2019a). Celastrol Alleviates Aortic Valve Calcification via Inhibition of NADPH Oxidase 2 in Valvular Interstitial Cells. JACC Basic Transl Sci. 5 (1), 35–49. 10.1016/j.jacbts.2019.10.004 32043019 PMC7000868

[B24] Liu H. Zhang X. Zhong X. Li Z. Cai S. Yang P. (2019b). Puerarin Inhibits Vascular Calcification of Uremic Rats. Eur. J. Pharmacol. 855, 235–243. 10.1016/j.ejphar.2019.05.023 31085237

[B25] Liu J. Liu C. Qian C. Abela G. Sun W. Kong X. (2021). Ginkgo Biloba Extract EGB761 Alleviates Warfarin-Induced Aortic Valve Calcification through the BMP2/Smad1/5/Runx2 Signaling Pathway. J. Cardiovasc. Pharmacol. 78 (3), 411–421. 10.1097/FJC.0000000000001082 34132687 PMC8440405

[B26] Lu Q. Xiang D. X. Yuan H. Y. Xiao Y. Yuan L. Q. Li H. B. (2014). Puerarin Attenuates Calcification of Vascular Smooth Muscle Cells. Am. J. Chin. Med. 42 (2), 337–347. 10.1142/S0192415X14500220 24707866

[B27] Luo M. Sun W. Bin Zhou Bin. Kong X. (2022). Emodin Alleviates Aortic Valvular Calcification by Inhibiting the AKT/FOXO1 Pathway. Ann. Anat. - Anatomischer Anzeiger 240, 151885. 10.1016/j.aanat.2021.151885 34958913

[B28] Pagnin E. Fadini G. De Toni R. Tiengo A. Calo L. Avogaro A. (2005). Diabetes Induces p66shcGene Expression in Human Peripheral Blood Mononuclear Cells: Relationship to Oxidative Stress. J. Clin. Endocrinol. Metab. 90 (2), 1130–1136. 10.1210/jc.2004-1283 15562031

[B29] Pawade T. Newby D. Dweck M. (2015). Calcification in Aortic Stenosis. J. Am. Coll. Cardiol. 66 (5), 561–577. 10.1016/j.jacc.2015.05.066 26227196

[B30] Phadwal K. Feng D. Zhu D. MacRae V. E. (2020). Autophagy as a Novel Therapeutic Target in Vascular Calcification. Pharmacol. Ther. 206, 107430. 10.1016/j.pharmthera.2019.107430 31647975

[B31] Qiu L. Z. Yue L. X. Ni Y. H. Zhou W. Huang C. S. Deng H. F. (2021). Emodin-Induced Oxidative Inhibition of Mitochondrial Function Assists BiP/IRE1α/CHOP Signaling-Mediated ER-Related Apoptosis. Oxidative Med. Cell. longevity 2021, 886581. 10.1155/2021/8865813 PMC8084644 33968299

[B32] Redza-Dutordoir M. Averill-Bates D. A. (2016). Activation of Apoptosis Signalling Pathways by Reactive Oxygen Species. Biochim. Biophys. Acta (Bba) - Mol. Cel Res. 1863 (12), 2977–2992. 10.1016/j.bbamcr.2016.09.012 27646922

[B33] Shi Y. Wang S. Peng H. Lv Y. Li W. Cheng S. (2019). Fibroblast Growth Factor 21 Attenuates Vascular Calcification by Alleviating Endoplasmic Reticulum Stress Mediated Apoptosis in Rats. Int. J. Biol. Sci. 15 (1), 138–147. 10.7150/ijbs.28873 30662354 PMC6329919

[B34] Song Y. Hou M. Li Z. Luo C. Ou J. Yu H. (2017). TLR4/NF-κB/Ceramide Signaling Contributes to Ox-LDL-Induced Calcification of Human Vascular Smooth Muscle Cells. Eur. J. Pharmacol. 794, 45–51. 10.1016/j.ejphar.2016.11.029 27876618

[B35] Song Z. Wei D. Chen Y. Chen L. Bian Y. Shen Y. (2019). Association of Astragaloside IV-Inhibited Autophagy and Mineralization in Vascular Smooth Muscle Cells with lncRNA H19 and DUSP5-Mediated ERK Signaling. Toxicol. Appl. Pharmacol. 364, 45–54. 10.1016/j.taap.2018.12.002 30529164

[B36] Sudo R. Sato F. Azechi T. Wachi H. (2015). 7-Ketocholesterol-induced Lysosomal Dysfunction Exacerbates Vascular Smooth Muscle Cell Calcification via Oxidative Stress. Genes Cell : devoted Mol. Cell. Mech. 20 (12), 982–991. 10.1111/gtc.12301 26419830

[B37] Tomayko E. J. Cachia A. J. Chung H. R. Wilund K. R. (2014). Resveratrol Supplementation Reduces Aortic Atherosclerosis and Calcification and Attenuates Loss of Aerobic Capacity in a Mouse Model of Uremia. J. Med. Food 17 (2), 278–283. 10.1089/jmf.2012.0219 24476222

[B38] Wang J. Qiu X. Xu T. Sheng Z. Yao L. (2019). Sclerostin/Receptor Related Protein 4 and Ginkgo Biloba Extract Alleviates β-Glycerophosphate-Induced Vascular Smooth Muscle Cell Calcification by Inhibiting Wnt/β-Catenin Pathway. Blood Purif. 47 (Suppl. 1), 17–23. 10.1159/000496219 30699436 PMC6518869

[B39] Xu L. Hu Z. Shen J. McQuillan P. M. (2015). Effects of Ginkgo Biloba Extract on Cerebral Oxygen and Glucose Metabolism in Elderly Patients with Pre-existing Cerebral Ischemia. Complement. therapies Med. 23 (2), 220–225. 10.1016/j.ctim.2014.12.009 25847559

[B40] Xu Y. Shen J. Ran Z. (2020). Emerging Views of Mitophagy in Immunity and Autoimmune Diseases. Autophagy 16 (1), 3–17. 10.1080/15548627.2019.1603547 30951392 PMC6984455

[B41] Xue Q. He N. Wang Z. Fu X. Aung L. Liu Y. (2021). Functional Roles and Mechanisms of Ginsenosides from Panax Ginseng in Atherosclerosis. J. ginseng Res. 45 (1), 22–31. 10.1016/j.jgr.2020.07.002 33437153 PMC7790891

[B42] Yang D. Wang T. Long M. Li P. (2020). Quercetin: Its Main Pharmacological Activity and Potential Application in Clinical Medicine. Oxidative Med. Cell. longevity 2020, 8825387. 10.1155/2020/8825387 PMC7790550 33488935

[B43] Yang P. Ling L. Sun W. Yang J. Zhang L. Chang G. (2018). Ginsenoside Rg1 Inhibits Apoptosis by Increasing Autophagy via the AMPK/mTOR Signaling in Serum Deprivation Macrophages. Acta Biochim. Biophys. Sinica 50 (2), 144–155. 10.1093/abbs/gmx136 29324976

[B44] Zhang C. Tan Z. Xie Y. Zhao Y. Huang T. Lu Z. (2019). Appoptosin Mediates Lesions Induced by Oxidative Stress through the JNK-FoxO1 Pathway. Front. Aging Neurosci. 11, 243. 10.3389/fnagi.2019.00243 31551758 PMC6737070

[B45] Zhang M. Brewer A. Schroder K. Santos C. Grieve D. Wang M. (2010). NADPH Oxidase-4 Mediates protection against Chronic Load-Induced Stress in Mouse Hearts by Enhancing Angiogenesis. Proc. Natl. Acad. Sci. 107 (42), 18121–18126. 10.1073/pnas.1009700107 20921387 PMC2964252

[B46] Zhang P. Li Y. Du Y. Li G. Wang L. Zhou F. (2016). Resveratrol Ameliorated Vascular Calcification by Regulating Sirt-1 and Nrf2. Transplant. Proc. 48 (10), 3378–3386. 10.1016/j.transproceed.2016.10.023 27931585

[B47] Zhao M. Xu M. Cai Y. Zhao G. Guan Y. Kong W. (2011). Mitochondrial Reactive Oxygen Species Promote P65 Nuclear Translocation Mediating High-Phosphate-Induced Vascular Calcification *In Vitro* and *In Vivo* . Kidney Int. 79 (10), 1071–1079. 10.1038/ki.2011.18 21368742

[B48] Zhou J. Li X. Y. Liu Y. J. Feng J. Wu Y. Shen H. M. (2021). Full-coverage Regulations of Autophagy by ROS: from Induction to Maturation. Autophagy, 1–16. Advance online publication. 10.1080/15548627.2021.1984656 PMC9225210 34662529

[B49] Zhou T. Wang Y. Liu M. Huang Y. Shi J. Dong N. (2020). Curcumin Inhibits Calcification of Human Aortic Valve Interstitial Cells by Interfering NF-κB, AKT, and ERK Pathways. Phytotherapy Res. : PTR 34 (8), 2074–2081. 10.1002/ptr.6674 32189385

